# Transcriptomic Profile of the Cockle *Cerastoderma edule* Exposed to Seasonal Diarrhetic Shellfish Toxin Contamination

**DOI:** 10.3390/toxins13110784

**Published:** 2021-11-05

**Authors:** Dany Domínguez-Pérez, José Carlos Martins, Daniela Almeida, Pedro Reis Costa, Vitor Vasconcelos, Alexandre Campos

**Affiliations:** 1 CIIMAR—Interdisciplinary Centre of Marine and Environmental Research, University of Porto, Rua General Norton de Matos s/n, Terminal de Cruzeiros do Porto de Leixões, 4450208 Matosinhos, Portugal; jmartins@ciimar.up.pt (J.C.M.); vmvascon@fc.up.pt (V.V.); 2 Department of Biological Sciences, Clemson University, Clemson, SC 29634, USA; danielaalmeida23@gmail.com; 3 IPMA—Instituto Português do Mar e da Atmosfera, Rua Alfredo Magalhães Ramalho, 6, 1495006 Lisbon, Portugal; prcosta@ipma.pt; 4 Biology Department, Faculty of Sciences, University of Porto, Rua do Campo Alegre, s/n, 4169007 Porto, Portugal

**Keywords:** DSTs, bivalves, RNAseq, biomarkers, Differentially Expressed Genes (DEGs), Portugal

## Abstract

Bivalves constitute an important source of proteins for human consumption, but some accumulate biotoxins such as diarrhetic shellfish toxins (DSTs), constituting a risk to human health. The cockle *Cerastoderma edule* is one of the most important species harvested in the Portuguese coast but also one of the most affected species due to recurrent DSTs exposure. However, little is known regarding the effects of the toxins produced by blooming dinoflagellates on *C. edule*. Herein, we explore the Differentially Expressed Genes (DEGs) of two tissues (gills and digestive gland) from wild cockles sampled in Portugal, through their whole transcriptomic response in two different seasons (exposed and not exposed to DSTs). The de novo transcriptome assembly returned 684,723 contigs, N50 of 1049, and 98.53% completeness. Altogether, 1098 DEGs were identified, of which 353 DEGs were exclusive for the digestive gland, 536 unique for the gills and 209 DEGs were common. Among DEGs were identified known DSTs-biomarkers including glutathione peroxidase, glutathione S-transferase, superoxide dismutase, cytochrome P450, ABC transporters, actin and tubulin-related proteins, Heat shock proteins and complement C1Q-like proteins. This study provides the first transcriptomic profile of *C. edule*, giving new insights about its molecular responses under different environmental conditions of DSTs exposure.

## 1. Introduction

Seasonal microalgae blooms constitute an ongoing concern due to their potential impacts on fisheries, seafood safety, tourism and other ecosystem services [1,2,3,4,5]. The high level of some marine biotoxins produced during these events, known as harmful algal blooms (HABs), can kill fish and other sea organisms on a large scale [6,7,8]. Currently, one of the most impactful marine biotoxins is the group of Diarrhetic Shellfish Toxins (DSTs) [6], comprising okadaic acid (OA) and analogous molecules named dinophysistoxins (DTX−1, −2 and −3) [9]. Indeed, DSTs outbreaks are the most abundant from recurrent HABs in Europe (North Atlantic) [8,10], as well as other temperate regions of the world such as Asia or South America [6]. The main source of DSTs in the marine trophic chain are species of the genus *Dinophysis,* but they can be produced by dinoflagellates belonging to the genera *Prorocentrum* as well [9].

DSTs are lipophilic toxins that can be filtered and accumulated in the adipose tissues by some marine organisms [11], being shellfish the main route of transmission to humans [12,13]. Shellfish constitutes an important source of protein for humans, but their consumption represents a food safety threat when contaminated with DSTs [13,14,15]. The ingestion of contaminated shellfish with DSTs can produce poisoning symptoms such as diarrhea, nausea, vomiting and abdominal pain [12,16]. In Portugal, for instance, there is an old tradition of eating shellfish, including some commercial species of bivalves like mussels, clams, scallops, oysters, and cockles [13]. However, DSTs shellfish contamination occurs every year, between April and October [2], resulting in the temporary banning of shellfish harvesting and trade [10].

The toxic effects of DSTs in humans mainly involve the inhibition of serine/threonine protein phosphatases 1 and 2A (PP1 and PP2A) [15,17], causing deregulation of several intracellular processes [13]. However, shellfish can accumulate a high amount of DSTs, mainly in the digestive gland [18,19], without known records of mortality associated with blooms of DST-producing dinoflagellates [13]. The mechanism underlying filter-feeding bivalves’ resistance to DSTs includes the removal of the toxins contained in non-digested microalgae cells and food materials through the production of feces and pseudo-feces, as well as activation of biotransformation and cellular excretion [9,18]. The toxin resistance at the molecular level has been associated with some antioxidant enzymes such as superoxide dismutase (SOD), catalase (CAT), and glutathione peroxidase (GPx) [20], phase-I and -II drug-metabolizing enzymes cytochrome P450 (CYP450) and glutathione s-transferase (GST) [21,22], and ATP-binding cassette (ABC) transporters (e.g., P-glycoprotein) [23,24,25].

Advances in OMICs approaches have corroborated these biomarkers in some bivalves contaminated with DSTs [9,13]. OMICs could contribute to identify the repertoire of the proteins involved in the dynamics of DSTs in shellfish, specificity, and selectivity of transforming enzymes to OA and DTX−1,−2 [13,20,26,27,28]. Nonetheless, a wide range of characterized transcripts, proteins, or metabolites in public databases are only available for some species, such as mussels and oysters. Indeed, the marine mussel *Mytilus galloprovincialis* [29] or oyster *Magallana gigas* (previously known as *Crassostrea gigas*) [30] are better represented, with fully sequenced genomes, transcriptome, and proteome characterization, while other commercial species like clams *Ruditapes decussatus* [31], the marine gastropod *Lobatus gigas* [32], tellin *Donax trunculus* and the cockle *C. edule*, remain poorly studied [13].

**Figure 1:**
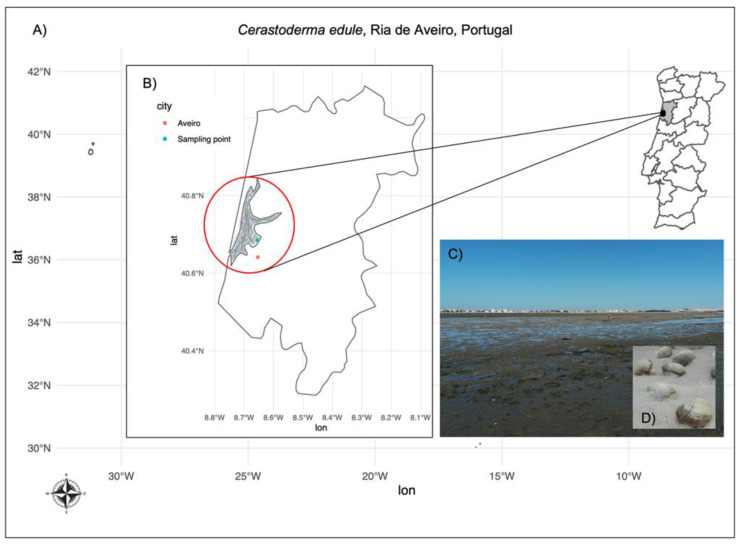
Shellfish harvesting area in Ria de Aveiro, Portugal. The composite figure displays in the background (**A**) the map of Portugal (*x*-axis: longitude, *y*-axis: latitude), highlighting the geographical localization of Ria de Aveiro, which is enlarged in panel (**B**), showing the coordinates of Aveiro city (red) and the sampling point (green). In the panel (**C**) is shown a picture of the harvesting area in Ria de Aveiro, during the low tide in the sampling point of (**D**) the cockles *Cerastoderma edule*.

It has been demonstrated that depuration rates of DSTs vary among species (cockles > oysters > scallops > clams > mussels) [9,18,33,34,35,36]. The cockle *C. edule* (Figure 1) is, within the group of bivalves, the species with the highest clearance rates. This unique depuration capacity is, however, poorly understood. *C. edule* constitutes an important commercial species in Portugal, which is still harvested and collected by the Portuguese population, using traditional techniques. Nonetheless, this is one of the most seasonally affected bivalve species by DST outbreaks in Portugal, mainly in the summer (Figure 1).

In this work we performed for the first time a transcriptomic analysis of two tissues from wild cockles sampled in Ria de Aveiro, Portugal (Figure 1), during two different seasons, namely during blooms of *Dinophysis* spp when high levels of DSTs are reached in cockles and in periods of no shellfish toxicity (exposed and not exposed to DSTs). Ria de Aveiro is a shallow lagoon located on the Portuguese Northwest Atlantic coast (Figure 1), and one of the major shellfish harvesting sites in this country [37]. This lagoon is repeatedly exposed to several HABs, including DSTs-producing dinoflagellates, that significantly impact the commercial harvesting of bivalves [37,38]. Thus, we sequenced the total RNA of two tissues: gills and digestive gland, using a paired-end RNA sequencing approach in an Illumina NovaSeq 6000 platform. These two organs are highly important to consider when examining the impacts of toxic algae exposure. The digestive gland is where the accumulation of biotoxins and their biotransformation processes mainly occurs, while the gills are in direct contact with water and toxic cells [18,39]. Some of these DEGs identified were consistent with known molecular biomarkers reported in the DSTs depuration process in bivalves. This study constitutes the first transcriptomic profile of *C. edule* exposure shedding new lights of the molecular response of this species in different environmental scenarios of DST exposure.

## 2. Results

### 2.1. DSTs Determination by LC-MS/MS in C. edule Sampled in Ria de Aveiro, Portugal

Herein, we performed a transcriptomic analysis of two tissues from wild cockles sampled in Ria de Aveiro (Figure 1), Portugal, in two different seasons of the year 2019. The DSTs content in the homogenate of samples from both seasons studied was determined via liquid chromatography with tandem mass spectrometry (LC-MS/MS). The quantity of DSTs was below the detection limit in the samples used as control (March 2019), which confirmed that such samples were not exposed to DSTs, whereas the exposed samples (May 2019) contained amounts over the limits permitted for human consumption, being 218.3 µg/kg for OA, 124.0 µg/kg for DTX2, which in terms of total OA equivalents corresponds to 292.8 µg/kg of total OA equivalent.

### 2.2. Illumina Sequencing, Transcriptome Assembly and Completeness

The total RNA of two tissues: gills and digestive gland, was extracted using an RNeasy Mini kit (Qiagen, Venlo, The Netherlands). RNA samples were conducted for paired-end RNA sequencing using an Illumina NovaSeq 6000 platform (Illumina, Inc., San Diego, CA, USA) at Macrogen (Macrogen, Inc., Seoul, Korea). The total RNA sequencing delivered around 41.5 reads per sample with 151 bp of read length, accounting for more than 6.2 billion bases (Table 1). Most of the sequenced reads were identified with high accuracy, with approximately 98% showing a Phred quality score (i.e., ratio of bases that expresses the accuracy of each nucleotide) over 20 (Q20: corresponding to 99% base call accuracy), and around 94% of reads with Phred score over 30 (Q30: corresponding to 99.9% base call accuracy) (Table 1). After adapter trimming, high quality reads were conducted for de novo transcriptome assembly using Trinity v2.10.0.

Around 93% of the pre-processed reads comprising 504,019,879 of bases, were aligned in the de novo assembly. Altogether, we obtained an assembled transcriptome with 684,723 transcripts and 361,460 genes by combining all four samples into a single transcriptome, of which 684,576 transcripts showed length over 200 bp (Table 1). The de novo assembly generated showed a contig length (N50) of 1094 bp, average contig length of 736.09 bp, with a minimum and maximum contig length of 200 bp and 42,164 bp, respectively (Table 1). The assembly completeness revealed that 98.53% of the BUSCO groups have complete gene representation (single-copy or duplicated), while 0.84% are only partially recovered, and 0.63% are missing (Table 1). The number of duplicated BUSCOs were reduced from 68.97% to 29.56% after clustering analyses (Figure S1), resulting in 402,961 number of clusters at 90% identity (Table 1). The transcriptomic project has been registered with the BioProject database under BioProject ID: PRJNA739261 (http://www.ncbi.nlm.nih.gov/bioproject/739261). It is noteworthy, that 147 remaining transcripts with length below 200 bp, and additional 4930 transcripts, detected as putative contaminants were removed during NCBI transcriptome submission. This filtered Transcriptome Shotgun Assembly project containing 679,646 contigs has been deposited at DDBJ/EMBL/GenBank under the accession GJGL00000000. All statistics and additional data of the total RNA sequencing, assembly, and clustering are summarized in Table 1, DATASET S1, as well as in the data availability section.

### 2.3. Open Reading Frame Prediction and Annotation

The 402,961 clusters obtained with 0.9 of sequence identity were used for the open reading frames (ORFs) prediction using the TransDecoder v5.5.0., considering a minimum length of 100 amino acids. Overall, 48,107 protein coding sequences (CDS) were identified with homology to known proteins via Pfam searches as the best/longest isoform per gene (Table 1). A total of 21,788 CDS were complete proteins, corresponding to 45%, whereas around 33% were partial sequences, and 21% internal sequences (Figure S2, DATASET S2).

These 48,107 ORFs were CloudBlasted against the metazoans section of the non-redundant (nr) protein database from NCBI and UniProtKB/Swiss-Prot protein database [40], using CloudBlast option with the BlastX program setting a cut-off E-Value 1.0E−3 [41]. A total of 37,184 transcripts had hits against metazoans and 362 against non-metazoans species within the nr database. On the other hand, 26,968 transcripts showed significant homology to metazoans species in UniProtKB/Swiss-Prot protein database, and 22 transcripts with non-metazoans species, of which 20 hits were common to non-metazoan matches found in the nr database. Overall, 37,262 transcript sequences corresponding to 77.5% had blast hits, whereas 10,845 corresponding to 22.5% (Table 1) remained without hits.

Besides, 31,848 (66.2%) of these 48,107 ORFs were also annotated with eggNOG-Mapper 1.0.3 with EggNOG 5.0.0 [42]. Moreover, the corresponding GO annotation was performed by combining the existing GO terms (Blast hits mapping/GO annotation, EggNOG, InterProScan) and KEGG pathways [43]. In total, 21,799 (45.3%) transcripts were functionally annotated by Gene Ontology (Table 1).

It is noteworthy that our samples were retrieved from the wild, thus, putative contaminants were detected (Figure S3) and carefully filtered before pairwise differential expression analyses. Indeed, 611 transcripts out of the 48,107 predicted ORFs (as best isoforms), were filtered out considering Blast hits description against non-metazoans section, eggNOG best Taxa Level, and putative contaminants automatically detected by the NCBI/TSA submission system. The remaining 47,496 ORFs encoded by 33,665 genes were then used for differential expression analyses. The list of these 47,496 ORFs and the list of putative contaminants with their corresponding annotation (e.g., Blast Top-Hits, GO annotation and eggNOG annotation) can be found in Tables S1 and S2, respectively.

### 2.4. Differential Expression Analyses and Enrichment

**Figure 2:**
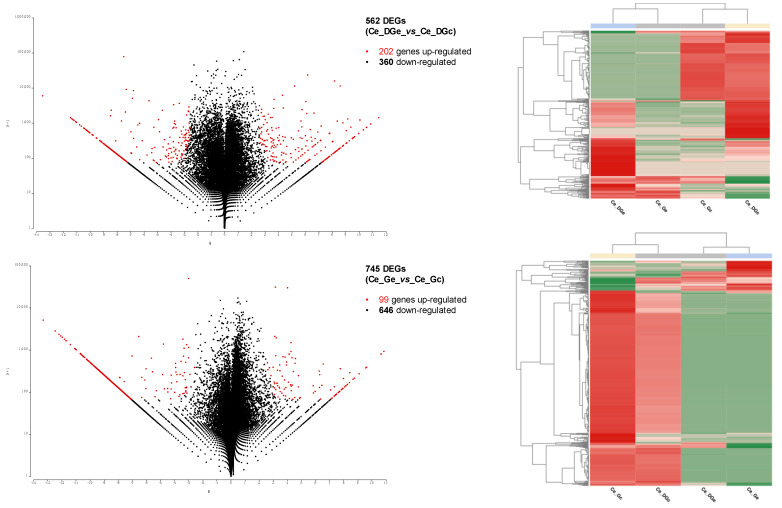
Pairwise Differential Expression results using NOISeq R/Bioc package (*p* < 0.01). The figure shows a scatter plot showing the log-fold change (M) and the absolute value of the difference in expression between conditions (D), where D values are displayed in a log scale. The Differentially Expressed Genes (DEGs) are depicted as red dots. The corresponding heat map of DEGs (Z-score values) from each condition tested (in columns) are depicted: Ce_DGe_*vs*_Ce_DGc, in the top panel corresponding to Digestive Gland (DG) samples, and Gills (G) in the bottom (Ce_Ge_*vs*_Ce_Gc). The contrast condition and reference/control samples are shown as Ce DGe/Ce Ge and Ce DGc/Ce Gc, respectively.

All transcripts encoded by the 33,665 *C. edule* coding genes (related with the 47,496 ORFs) were used as the template to calculate gene expression. In total, 141,638 transcripts were retrieved from the de novo transcriptome assembly and then used to map the pre-processed raw data to create the count tables for each pairwise comparison. Considering the distribution of counts across libraries (Figure S4), some feature/isoforms were filtered on a count-per-million basis (CPM). Altogether, 31,772 genes were retained after counts filtering and then considered for pairwise differential expression analyses in the digestive gland, while in the gills were used 30,861 features. The differential expression analyses for the digestive gland resulted in 562 DEGs, of which 202 genes were up-regulated and 360 down-regulated, whereas in the gills were found 745 DEGs, being 99 up-regulated and 646 genes down-regulated (Figure 2).

**Figure 3:**
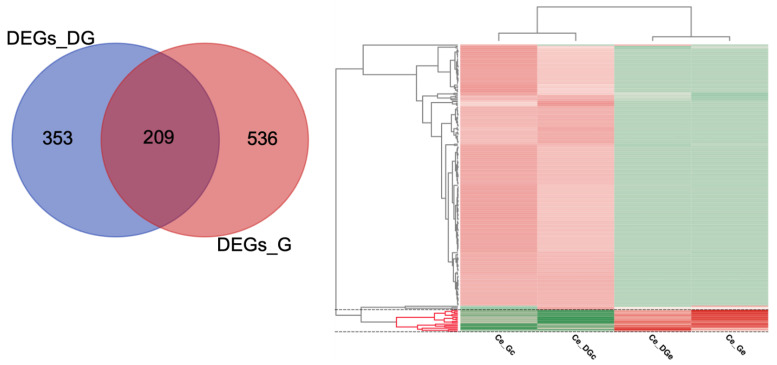
Venn Diagram of the Differentially Expressed Genes (DEGs) and heat map of the shared DEGs between pairwise comparisons. The figure shows in the left panel unique and overlapped DEGs between the tested conditions Ce_DGe_*vs*_Ce_DGc, and Ce_Ge_*vs*_Ce_Gc, corresponding to Digestive Gland (DEGs_DG) samples, and Gills (DEGs_G). The contrast condition and reference/control sample are shown in the columns of the heat map (right panel) as Ce_DGe/Ce_Ge and Ce_DGc/Ce_Gc, respectively. The heat map depicts the corresponding expression (Z-score values) of the 209 common DEGs found, of which a cluster of up-regulated genes in the exposed condition, comprising 16 transcripts highlighted (in the bottom between dashed lines). Venn diagram was drawn with an online free tool, available at the webserver of the Bioinformatics and Evolutionary Genomics Center (BEG/Van de Peer Lab site, Ghent University, Belgium, http://bioinformatics.psb.ugent.be/webtools/Venn/accessed on 15 July 2021).

In total, 1098 unique/non-redundant DEGs were obtained in both pairwise comparisons, 209 DEGs were common in both tissues, being 353 DEGs exclusive for the digestive gland comparison, and 536 unique DEGs found in the gills (Figure 3). The pattern of DEGs expression is very similar among samples from different tissues of the same conditions. However, some genes show a tissue-dependent expression pattern (Figure 3). Indeed, among those 209 overlapped DEGs, 16 DEGs showed a similar up-regulated pattern in both exposed conditions, 189 DEGs showed a similar down-regulated pattern, and four DEGs were putatively non-condition-dependent (Figure 2, Tables S3 and S4).

**Figure 4:**
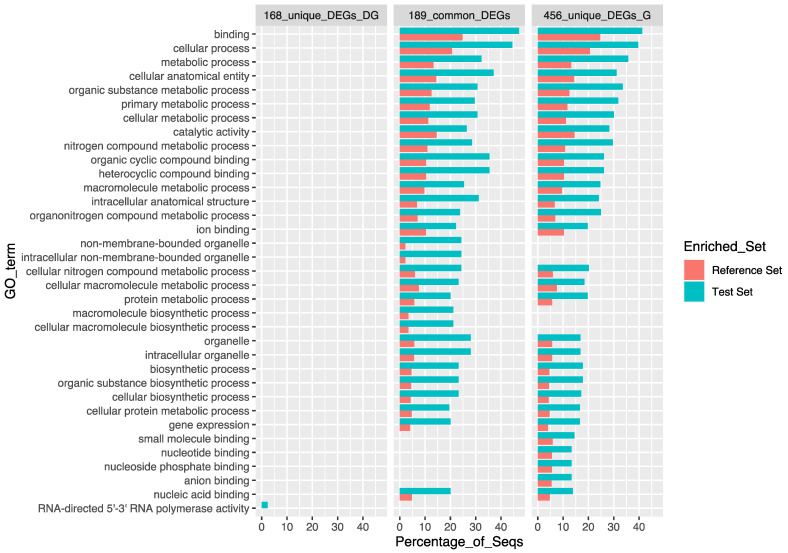
Enrichment analyses performed for the Differentially Expressed Genes (DEGs). The top-thirty enriched GO terms obtained with one-tailed Fisher’s exact test (FDR < 0.05) are represented by a stacked bar (in green) as the percentage of the GO terms found in the Reference Set (in red), for each subset of DEGs used as Test Set: 189 common DEGs with a similar down-regulated pattern identified in both condition exposed to DSTs (189_common_DEGs), unique down-regulated DEGs identified in the digestive gland (168_unique_DEGs_DG), and 456 unique down-regulated DEGs identified in the gills (456_unique_DEGs_G). The tested conditions in the pairwise analyses were Ce_DGe_*vs*_Ce_DGc and Ce_Ge_*vs*_Ce_Gc, corresponding to Digestive Gland (DEGs_DG) samples, and Gills (DEGs_G), where the contrast condition and reference/control samples were Ce_DGe/Ce_Ge and Ce_DGc/Ce_Gc, respectively.

Although some known stress and detoxing-DSTs related-genes were detected (Table 2), those 16 commons up-regulated DEGs in the exposed conditions are related to immune response (immunoglobulin-like and macro domain-like), enzymes, integral components of the membrane, unknown and/or poorly characterized elements (Figure 2, Tables S3 and S4). Thus, this subset did not enrich any GO term, as well as another subset of up-regulated DEGs (Table S3), by applying the hypergeometric distribution significance test (one-tailed Fisher’s exact test, FDR < 0.05). On the contrary, down-regulated DEGs showed significant differences in the enrichment analyses (Figure 4, Table S5), resulting in 110 enriched GO terms for the subset of 189 common DEGs with a similar down-regulated pattern identified in both conditions exposed to DSTs (189_common_DEGs), only one for those unique down-regulated DEGs identified in the digestive gland (168_unique_DEGs_DG), and 176 enriched GO terms for the 456 unique down-regulated DEGs identified in the gills (456_unique_DEGs_G).

Among the top-thirty enriched GO terms highlights: binding, cellular process, metabolic process, catalytic activity, intracellular anatomical, intracellular organelle, and biosynthetic process as common GO terms. Some terms related to non-membrane-bounded organelle and macromolecule biosynthetic process, are enriched in the subset of 189 common DEGs. In contrast, small molecule binding, nucleotide binding, nucleoside phosphate binding and anion binding, are exclusive for the subset of 456 unique DEGs found in the gill’s comparisons (Figure 4). The complete list of DEGs and the results of the Venn diagram, listing unique and shared DEGs among samples are provided in Tables S3 and S4, respectively. The enriched GO-terms found for each subset of DEGs, and the names of the related genes, can be found in Table S5. Additional files related to the gene expression and enrichment analyses can be found in the data availability section (DATASET S3–S5).

## 3. Discussion

### 3.1. Common DEGs and Known Biomarkers Facing DSTs

Herein, we identified 1098 unique/non-redundant DEGs, with 209 DEGs shared between comparisons (Figure 3), with a higher number of 745 DEGs in the gills (Ce_Ge_*vs*_Ce_Gc) compared to the 562 DEGs found in the digestive gland (Ce_DGe_*vs*_Ce_DGc) (Figure 2, Tables S3 and S4). A higher number of DEGs have been previously reported in the gills in similar studies assessed in the mussel *Mytilus galloprovincialis* [44]. However, this behavior is likely non-tissue dependent, since the number of DEGs and the Differentially Abundant/Expressed Proteins found have been variable at transcriptomic [28] and proteomic [26] levels, after mussels’ exposure to DSTs and their derivatives (i.e., OA, DTX1, DTX2 and DTX3). Nonetheless, some genes expression could be tissue-dependent once 353 DEGs were exclusive for the digestive gland comparisons, whereas 536 unique DEGs were found in the gills (Figure 3).

Although 98% of the common DEGs showed a similar expression pattern, only 7.65% (16 DEGs) were up-regulated in the exposed condition of both comparisons (Ce_DGe_*vs*_Ce_DGc and Ce_Ge_*vs*_Ce_Gc) (Figure 3, Table 2). This subset includes DEGs involved in immune response, enzymes, and poorly characterized genes/transcripts. Among the 16 commons up-regulated DEGs in the exposed conditions of both comparisons (Figure 3), highlights the transcript/gene TRINITY_DN2028_c0_g1 with homology to the CYP450 (Table 2, Table S3). The family of CYP450 enzymes with oxidoreductase/monooxygenase activity has been recognized as an important biomarker involved in metabolic detoxification, which has been found as up-regulated after DSTs exposure in shellfish [9,13,21,45,46]. In addition, another transcripts/gene TRINITY_DN9032_c0_g3 with homology to a CYP450 2U1-like (XP_021373153.1) from *Mizuhopecten yessoensis* was also found as up-regulated in the digestive gland (Table 2).

Likewise, DEGs involved in immune response were relatively abundant in the subset of 16 commons up-regulated DEGs, denoting a possible immunological response of the clam to the exposure and ingestion of toxic alga and/or parasites (Table 2). The transcript/genes TRINITY_DN909_c0_g1, showed homology to neural cell adhesion molecule 1-like from the oyster *Crassostrea gigas* (XP_034299592.1) and TRINITY_DN802_c0_g1 with nephrin-like from *M. yessoensis* (XP_021372895.1), which contain immunoglobulin-like domains. Besides, another DEGs (TRINITY_DN3944_c0_g2), showed an Immunoglobulin-like domain, identified throughout IPS accession (IPR007110). These domains are often involved in interactions, commonly with other Ig-like domains via their β-sheets and can be found in different types of receptors like T-cell antigen receptors, cell adhesion molecules, MHC class I and II antigens, as well as the hemolymph protein hemolin [47,48,49,50,51]. Noteworthy, one protein can contain more than one of these types of Ig-like domains, for instance: Immunoglobulin C-2 Type, Ig_3 domain and cytokine receptor motif NF3 (fibronectin type 3 domain), C1-set (constant-1; IPR003597), C2-set (constant-2; IPR008424) and I-set (intermediate; IPR013098) [52].

Interestingly, hemolin, formerly known as bacteria-induced protein P4 [53], belongs to the immunoglobulin superfamily, considered one of the first hemolymph components to bind to the bacterial surface [53]. This protein has been mainly characterized in insects [54,55,56,57] within the major bacteria-inducible hemolymph protein [57], but hemolin also occurs in crustaceans and mollusks [58,59] as part of the host defense mechanisms [60]. An hemolin-related molluscan defense molecule, with five Ig-like domains was identified as down-regulated in *Lymnaea stugnalis*, when infected by the schistosome parasites [59]. However, this report is inconsistent with our findings, since most of the DEGs related with parasites were down-regulated (Figure 2, Table 2 and Table S3), suggesting less abundance in the exposed condition, where hemolin showed higher expression (Figure 3, Table S3). Only the DEGs TRINITY_DN842_c2_g1 within this subset, with homology to a C-Jun-amino-terminal kinase-interacting 4-like isoform X6 from *Schistosoma japonicum* (TNN04577.1) was up-regulated. Although the population of parasites detected in *C. edule* is variable among seasons in Ria de Aveiro, their abundances lack significant differences [61]. Likewise, a higher mean temperature of previous months can negatively affect the prevalence of parasites in *C. edule* [62]. In addition, two transcripts/genes matching to heat shock proteins HSP 90 (HSP90) from parasites, were down-regulated, being one of them commonly found within the 189 shared DEGs in both tissues (Table 2). In general, the probability of detecting a trematode-related genes as down-regulated within DEGs suggests a less infestation of those parasites in the exposed condition (sampled in May), than control (March).

In addition, other elements related to immune response were identified among DEGs (Table 2). Although some of them have been previously identified in response to DSTs in bivalves [13], others could potentially belong to parasites, as well as DEGs involved in the transcription regulation (Table 2). However, any of the matched parasites have been reported within the list of species that use *C. edule* as their first and/or second intermediate host [61,62]. Likewise, the best-hit species distribution could be biased by the annotation method using the blastX program (homology-based), and the availability of homologues sequences in the public database. Some of these DEGs involved in immune response include five transcript/genes related to complement C1q (Table 2). The complement components C1q, such as mgC1q83 and mgC1q29 were significantly down-regulated in the transcriptomic analyses of the digestive gland of the mussel *Perna viridis*, after 6-h exposure to DSTs-producing dinoflagellate *P. lima* [63]. In contrast, we found four transcripts differentially expressed in the digestive gland, of which two DEGs (TRINITY_DN10313_c0_g2 and TRINITY_DN45102_c0_g1) were down-regulated, and two (TRINITY_DN1050_c0_g1 and TRINITY_DN1909_c0_g2) up-regulated (Table 2). Besides, we also found a gene/transcript (TRINITY_DN14010_c0_g1) with homology to a C1q domain-containing protein MgC1q83 from *M. galloprovincialis* (CBX41732.1) in the gills (Table 2). This DEG was significantly down-regulated in the gills, having the same blast-hit (CBX41732.1) of the DEGs reported as a C1q genes down-regulated in the digestive gland of *P. viridis* [63].

Herein, we found that both organs studied can produce these components under immune stress conditions, but with some differences regarding the isoform distribution and the up-down-regulation pattern among DEGs between conditions/tissues (Table 2). The C1q family is involved in various known immune responses, such as pathogen recognition, activation of the complement system, mediating cell migration, and apoptotic cell clearance [64,65]. In the Mediterranean mussel *M. galloprovincialis*, MgClq plays a role in the recognition of pathogens as part of their innate immune response [66]. Thus, considering their important role and versatility [63,65,66], the expression of some transcripts/isoforms could be tissue-dependent, since any of the DEG related to C1q complement were shared among conditions, being negatively affected in some cases such as MgC1q83 (CBX41732.1), but also activated in the digestive gland (Table 2).

In addition, other biomarkers involved in the antioxidant system and metabolic detoxification were detected (Table 2). Three down-regulated DEGs (two in the digestive gland and one in the gills) showed homology to SOD (Table 2). The transcripts/gene TRINITY_DN7416_c0_g1 with homology to a GST Mu 3 (XP_006260118.1) was down-regulated in the gills, as well as the DEGs TRINITY_DN54684_c0_g1 matching to a GPx enzyme (Table 2). On the contrary, two DEGs (TRINITY_DN1482_c1_g1 and TRINITY_DN8990_c0_g1) functionally related to the glutathione catabolic process, glutathione hydrolase activity and γ-glutamylcyclotransferase activity, were up-regulated in the digestive gland. Similarly, two DEGs related to ABC transporters’ family genes were up-regulated in the digestive gland (Table 2). Altogether, these antioxidant elements (such as SOD, CAT, GST, GPx), as well as detoxifying enzymes like CYP450, and the ABC transporters family, play an active role in the first line of defense against DSTs in bivalves’ mollusks [20,21,22,23,24].

Previous studies also found GST as down-regulated, suggesting a main role during short-term exposure of DSTs, as proposed for ABC transporters [63]. However, other GST-related/Phase II detoxification enzymes were up-regulated in the digestive gland as well as some members of the ABC transporters family genes. These group of genes seem to remain up-regulated/activated in a longer exposure period to catalyze the conjugation of glutathione to the DSTs and derivates for its further elimination through the ABC transporter (ABCB10) system and/or multidrug resistance-associated protein (ABCC1, ABCG) [63,67]. Nonetheless, the up-regulation of some ATP-binding cassette (ABC) transporters family genes could be relevant in the mechanism of bivalve’s resistance in a longer exposure period, since they can play a variety of roles in xenobiotic transmembrane transporter activity and detoxification.

Components of the cytoskeleton, microtubule constituents, and actin-related genes were also affected (Table 2, Table S3). Indeed, a total of seven DEGs were found as downregulated in both tissues, comprising actin-1 isoform X2, tubulin α chain, actin depolymerizing factor, VChain V/β-actin, and actin β/γ 1 (Table 2). Damage in the cytoskeleton and/or destabilization in its elements like actin and tubulin have been corroborated as truthful markers of DST effects in bivalves [23,63,67]. Indeed, TUBA1C and TUBB1 were significantly down-regulated in the gills of the mussel *P. viridis* after a short-time exposure of *P. lima* [67]. The histological study revealed the branchial filament structure completely deformed, and the cells atrophied into strips with irregular arrangement during the short-term exposure. However, the negative effect to the gill decreased, and the gill was restored after longer exposure (96 h), associated with a significant increase in the expression of TUBA1C and TUBB1 transcripts [67]. This behavior was not observed in this study since those genes TUBA1C and TUBB1 were not detected within our list of DEGs. However, the down-regulation of cytoskeleton-related genes in the exposed condition can be explained by the recovery after the long-term DSTs exposure.

### 3.2. Enriched Pathways and Degs Involved

The lack of GO terms associated with these 16 DEGs up-regulated genes may difficult the unbiased detection of enriched pathways in this subset. In fact, only seven genes/transcript (43.75%) had GO annotations, and four DEGs (TRINITY_DN961_c0_g1, TRINITY_DN9102_c0_g1, TRINITY_DN768_c3_g1, TRINITY_DN13635_c0_g1) lacked any Blast hits, IPS results, and Pfam/conserved domains (Figure 2, Tables S3 and S4).

Similarly, only one GO term RNA-directed 5′-3′ RNA polymerase activity (GO:0003968) was enriched in the subset of the 168 unique down-regulated DEGs found in the digestive gland (Figure 4, Table S5). Four transcript/genes TRINITY_DN71766_c0_g1, TRINITY_DN126419_c0_g1, TRINITY_DN7379_c0_g1 and TRINITY_DN18481_c0_g1, included within the test set of DEGs are related to flavirus-like protein and/or polyprotein/polyprotein-4 (Table S3). These transcripts/genes show domain of enzymes (RNA-dependent RNA polymerase, RdRp), which are essential protein encoded in the genomes of all RNA containing viruses with no DNA stage [68,69]. These transcripts/genes are putatively derived from the non-exposed samples (used as control, sampled in March), containing retroviruses, seemingly single stranded RNA viruses (Figure S5).

Apart from this unique over-represented GO term, the other 189 shared down-regulated DEGs from the digestive gland, enriched most of the GO terms found in the subset of 456 unique down-regulated DEGs from the gills (Figure 4, Table S5). Indeed, many overrepresented GO terms were found in both subsets of shared DEGs (168_unique_DEGs_DG), and unique down-regulated DEGs from the gills (456_unique_DEGs_G) (Figure 4, Table S5). Although most of the over-represented pathways are common in the abovementioned subsets of DEGs (189 shared down-regulated DEGs and 456 unique down-regulated DEGs in the gills), some GO terms are exclusive for each group (Figure 4).

For instance, some enriched GO terms like binding (GO:0005488), cellular process (GO:0009987), metabolic process (GO:0008152), among others, display a similar enrichment pattern in the subsets of the common down-regulated DEGs and unique from the gills (Figure 4). Their absent in the exclusive down-regulated DEGs in the digestive gland (168_unique_DEGs_DG), suggest that both tissues can play similar function in non-exposed/exposed DSTs condition (without excluding other environmental factors (e.g., light, temperatures, parasites). Nonetheless, the gills possess some additional DEGs that can play similar roles related to those GO terms, except for non-membrane-bounded organelle (GO:0043228), intracellular non-membrane-bounded organelle (GO:0043232), macromolecule biosynthetic process (GO:0034645) and cellular macromolecule biosynthetic process (GO:0034645). Noteworthy, the GO terms small molecule binding (GO:0036094), nucleotide binding (GO:0000166), nucleoside phosphate binding (GO:1901265) and anion binding (GO:0043168), are exclusively over-represented in the subset of the 456 down-regulated DEGs in the gills (456_unique_DEGs_G) (Figure 4).

As expected, a high percentage of the down-regulated DEGs contained in those common 23 GO terms (Figure 4, Table S6), are redundant among those GO terms (Table S6). Besides, this redundancy is also observed when overlap the GO terms associated with the resulting DEGs groups from the Venn Diagram (Table S6). The “pleiotropic” behavior of the common DEGs may be related with the active role played in several basic function of great relevance to response the environmental changes such as DSTs blooms. Indeed, some terms like ribosome, transcriptions, HSPs, cytoskeleton, histones and glycolysis were more frequent among the DEGs in this subset (Table S6). Similar redundancy and functional roles were observed with the GO terms found in the subsets of common downregulated DEGs, which are also absent in the subset of unique DEGs in the digestive gland and gills (Figure 4). Likewise, the uniquely over-represented GO terms in the subset of the 456 down-regulated DEGs in the gills (small molecule binding, nucleotide binding, nucleoside phosphate binding and anion binding), are mainly involved in similar functions and putatively related to parasites as well (Tables S3 and S5).

## 4. Remarks

Herein, we performed a transcriptomic analysis of two tissues (gills and digestive gland) from wild cockles sampled in Ria de Aveiro, Portugal, in two different seasons (with and without DSTs). We found that some biomarkers previously reported among known stress and detoxing-DSTs related-genes were identified within DEGs such as: GPx, GST Mu 3, SOD and CYP450 (involved in antioxidant system), ABC transporter family members (metabolic detoxification/xenobiotic transmembrane transporter activity), apoptosis regulator protein 1 and CREB-binding protein (transcription regulators), actin 1 isoform X2, actin β/γ 1 and tubulin α chain (cytoskeleton, actin-related proteins), HSP90, and complement C1Q-like proteins (immune responses).

The detection of some known biomarkers involved in metabolic detoxification was consistent with previous studies, being CYPP540 up-regulated in the exposed conditions of both tissues analyzed, and two members of the ABC transporters family genes up-regulated in the digestive gland. Other biomarkers, such as enzymes, cytoskeleton components, and C1q component mgC1q83 showed a similar pattern as previously described. However, we found that C1q family gene expression could be more complex than previously known, seemingly tissue-dependent considering the isoforms diversity, distribution, and their down and up-regulation pattern. The isoforms of the C1q components found within DEGs should be particularly further studied since this C1q is recognized as the target recognition protein of the classical complement pathway concerning various immune responses. Although some of the DEGs found have been previously reported in shellfish after DST exposures, appearing as consistent biomarkers, their expression should not be only associated with DSTs, since we analyzed environmental samples. For instance, some enzymes in the oxidative stress response can be affected by high temperatures in mussels [70,71]. Similarly, the differential expression of the genes involved in the immune response can be induced by the DSTs concentration, but also influenced by the presence of parasites, bacteria, symbionts, and viruses. Noteworthily, some of these biomarkers ascribed to bivalves’ responses facing DST exposures involved in immune responses, antioxidant system, regulation of transcription, detoxification could also be found differentially expressed in both *C. edule* and putative parasites. This behavior in which parasites have been well represented within the annotation of mussels’ transcriptome was previously observed [63]. Hence, some genes should be carefully reviewed to avoid false conclusions whether some DEGs/known biomarkers belong to parasites or to the host.

To the best of our knowledge, this study provides the first transcriptomic profile of *C. edule*, giving new insights about its molecular responses facing DSTs exposure in a different environmental condition. Besides, the data generated and publicly shared constitute a valuable resource for bioinformatics analyses to identify sequences signatures in bio-transforming enzymes/proteins, allowing the design of specific primers for further in vitro assays such as RT-PCR targeting known and putative novel DSTs-biomarkers. Moreover, the transcriptomic profile can be used to create a custom protein database for high-throughput proteogenomic analyses to evaluate and/or validate the DEGs/DSTs-biomarkers expressions at the proteomic level. Altogether, our findings and the data provided could help to improve or address new depuration in the shellfish industry.

## 5. Materials and Methods

### 5.1. Sampling, RNA Extraction and RNASeq

Specimens of *C. edule* were retrieved from the wild in Ria de Aveiro (Canal de Mira, coordinates: 40.62713495410355, -8.739629051658452) in two different seasons of the year 2019 for comparison purposes. Conditions were established as without DSTs (control) and contaminated (exposed to DSTs) samples considering the warnings reports released by the Instituto Português do Mar e da Atmosfera (IPMA) (available at http://www.ipma.pt/pt/bivalves/index.jsp accessed on 20 March 2019, and 18 May 2019). The control samples corresponding to the season without risk of contamination with DSTs were collected on 22 March 2019. On this first sampling day, and according to IPMA phytoplankton records, the abundance (cells/L) of HAB species was below detection limits. Contaminated samples were sampled on 20 May 2019, when DSP toxin-producing species abundance levels attained 360 cells/L. Amnesic shellfish poisoning (ASP) toxin producers and yessotoxin-producing organisms were also detected, although in a much lower relative amount (taking into account regulated values).

Firstly, cockles were hand dredged during low tide at the harvest location in Ria de Aveiro, and then the specimens were transported in ice to the lab for subsequent tissues sampling. Small pieces (1 cm^3^) of gills (G) and digestive gland (DG) from five individuals of both conditions (control and treated) were pooled per tissue and condition, stabilized in RNAlater solution, and stored at 20 °C until used. The soft resting parts of the specimens were pooled and conducted to LC-MS/MS analysis for dinophysistoxins quantification.

### 5.2. DSTs Quantification by LC-MS/MS Analyses

To confirm the amount of DSTs such as okadaic acid (OA) and its analogs dinophysistoxins (DTXs) reported by IPMA in Ria de Aveiro (2019), the remaining soft-parts from the same *C. edule* specimens used for RNAseq, were pooled, and conducted to liquid chromatography with tandem mass spectrometry (LC-MS/MS), following the Standardized Operating Procedure (SOP) of the European Reference Laboratory for Marine Biotoxins (EURLMB) for the determination of marine lipophilic biotoxins in bivalve mollusks [72].

As previously described [26], a 2 g aliquot of cockle tissue homogenate was extracted with 9.0 mL MeOH 100% and homogenized with a Polytron mixer (Kinematica, Malters, Switzerland). The mixture was centrifuged at 2000× *g* for 10 min. The supernatant was then collected into a 30 mL tube and the pellet was re-extracted with 9.0 mL MeOH by vortexing for 2 min. After centrifugation, supernatants were combined and made up to 20 mL with MeOH. A 2.5 mL aliquot was the raw methanolic extract was transferred to 10 mL glass tube for alkaline hydrolysis to converting the 7-O-acyl ester derivatives (DTX3) into their parent toxin. The hydrolysis was started by adding 313 µL of 2.5 M NaOH and heated at 76 °C for 40 min in a heating block. The reaction was then neutralized with 313 µL of 2.5 M HCl. After filtered through 0.2 µm syringe filter 5 µL were injected to the LC-MS/MS system.

The LC-MS/MS analysis was performed using on in 1290 Infinity chromatograph (Agilent Technologies, Waldbronn, Germany) coupled to an Agilent 6470 triple quadrupole mass spectrometer. The chromatographic separation was conducted with a Zorbax SB-C8 RRHT column (2.1 × 50 mm, 1.8 µm), protected with a guard column (2.1 × 5 mm,1.8 µm). The mobile phase A was water with 2 mM ammonium formate and 50 mM formic acid, and the mobile phase B was 95% acetonitrile with 2 mM ammonium formate and 50 mM formic acid. An elution gradient at a flow rate of 0.4 mL/minute was used as follows: 0–3 min, gradient from 88 to 50% eluent A; 3–6.5 min gradient 50 to 10% eluent A; 6.5–8.9 min 10% eluent A; 8.9–10 min, gradient 10 to 88% eluent A. The detection of the toxins was carried out in multiple reaction monitoring (MRM) acquisition mode. Two MRM transitions were monitored in negative polarity, one for quantification and the other for confirmation. OA and DTX2: *m/z* 803 > 255 and *m/z* 803 > 563; DTX1: *m/z* 817 > 255 and *m/z* 817 > 563. A matrix match calibration with five standard solutions ranging in concentration levels from 2.0 to 22.0 ng/mL with a correlation > 0.990 was set up for quantification using certified OA, DTX1 and DTX2 reference standards purchased from CIFGA (Lugo, Spain).

### 5.3. RNA Extraction and Illumina Sequencing

Preserved samples in RNAlater solution were transposed to 2 mL tubes pre-filled with ceramic beads (Precellys^®^ Ceramic kit 1.4 mm, Bertin Technologies, Montigny-le-Bretonneux, France) and then homogenized in homogenization buffer (RLT buffer) using an automatic bead-based homogenizer (Bertin Technologies, Montigny-le-Bretonneux, France) with a custom program (speed: 5500 RPM at 0 °C; 2 × 20 s cycles; 30 s pause). The supernatant of the two tissues (G and DG) and both conditions, containing a pool from the five biological replicates, were recovered, and conducted for total RNA extraction using Qiagen’s RNeasy Mini kit (Venlo, The Netherlands) according to the manufacturer’s instructions.

Total RNA concentration and relative quality were determined photometrically with a DeNovix DS-11 spectrophotometer (DeNovix Technologies, Wilmington, DE, USA), and RNA integrity was evaluated with the 2100 Bioanalyzer (Agilent Technologies, Santa Clara, CA, USA). Four samples, corresponding to control samples (without toxins)—gills (Gc), digestive gland (DGc)—and contaminated samples (exposed or contaminated with shellfish toxins)—gills (Ge) and digestive gland (DGe)—were submitted to Macrogen, Inc. (Seoul, South Korea) for total RNA sequencing using Illumina TruSeq Stranded Total RNA Library Construction with Ribo-Zero + NovaSeq 6000.

### 5.4. Transcriptome Assembly and Completeness Assessment

The RNA sequencing on the aforementioned Illumina platform 150 bp (base-pair) paired-end (PE) resulted in around 41 million reads for each sample transcriptome. Transcriptomic and downstream analyses were performed with a set of bioinformatics tools incorporated in the OmicsBox v1.4.11 [41]. Firstly, the quality of the raw reads was assessed using the FastQC software v0.11.8 (http://www.bioinformatics.babraham.ac.uk/projects/fastqc/accessed on 13 January 2021) [73]. Then, the original FASTQ files were pre-processed using Trimmomatic v0.38 [74]. The adapters were trimmed for each sample individually, low-quality bases and low-quality reads removed, and only reads longer than 36 bp were retained for further analyses, as previously described [75]. Likewise, the filtered reads across all four samples were combined into a single dataset [75], and de novo assembled with Trinity v2.10.0 [76], using three as the minimum kmer coverage in the strand-specific mode. The assembly quality and completeness were assessed with the Benchmarking Universal Single-Copy Orthologs (BUSCOs, BUSCO v5) [77], searched for metazoans orthologues in transcriptome mode with a cut-off e-value 1.0E-3 on OrthoDB v10 [78].

### 5.5. Open Reading Frame Prediction and Transcript Abundance Estimation

Due to the lack of a reference genome for *C. edule*, the resulting de novo transcriptome assembly was used as a reference to perform the transcript expression analysis [75], using individual tools embedded in the Trinity differential expression module [79], and already included in OmicsBox [41]. In order to reduce sequence redundancy, the de novo assembled transcriptome was first submitted to clustering analyses using CD-HIT 4.8.1 [80,81], setting 0.9 as the sequence identity threshold. Then, the clustered transcriptome was scanned for coding regions by TransDecoder v5.5.0 (http://transdecoder.sf.net accessed on 3 February 2021) [79,82], and all six-frame translations were filtered for a minimum length of 100 amino acids for open reading frames (ORFs). Simultaneously, the resulting ORFs were scanned for homology to known proteins via Pfam searches (PFAM 32, HMMER 3.2.1) [83,84], and only the best isoform per gene was retained, considering as priority the homology to known proteins domain versus the transcript length [79,82]. The corresponding coding genes for the best isoforms and all their transcript isoforms were used as reference transcriptome templates for the expression analyses. The original pre-processed reads (clean reads without adapters) were mapped back against the reference transcriptome on a per-sample basis using Bowtie2 v2.3.4.1 [85], followed by calculation of abundance estimates using RSEM [86]. The resulting count tables obtained were used for the Pairwise Differential Expression Analysis (No Replicates) tool, based on the Bioconductor software package NOISeq v2.30.0 [87,88], suited to compare samples from two experimental conditions without biological replicates. The genes without counts across libraries were filtered on a count-per-million basis (CPM), and five technical replicates were simulated per sample. The data were normalized using the trimmed mean of M-values (TMM) normalization method, which was corrected for library size and reduced RNA compositional effect [89]. Finally, the Differential Expressed Genes (DEGs) were determined (*p*-value 0.01) between samples exposed to DST (contrast condition) versus samples without toxins (control conditions) from both tissues studied (digestive gland: Ce_DGe_versus_Ce_DGc and gills: Ce_Ge_versus_Ce_Gc).

### 5.6. Functional Annotation and Enrichment

The best isoforms per coding gene obtained from the clustered transcriptome, previously analyzed against Pfam, were then characterized based on sequence similarity against public databases. Firstly, these transcripts (best isoforms) were searched against the metazoans section of the non-redundant (nr) protein database from NCBI (nr database: ftp://ftp.ncbi.nlm.nih.gov/blast/db; accessed on 11 May 2021) and UniProtKB/Swiss-Protproteindatabase (accessed on 12 May 2021) [40], using CloudBlast option with the BlastX 2.10.0+ program setting a cut-off e-value 1.0E-3 [41]. The transcripts without hits were retrieved and CloudBlasted against the same protein database but excluding metazoans. Protein signatures were also obtained from the InterPro member databases (i.e., Pfam, PROSITE, PRINTS, ProDom, SMART, TIGRFAMs, PIRSF, SUPERFAMILY, Gene3D, and PANTHER) using the InterProScan (v5.51–85.0) software [43]. All annotation and the corresponding gene ontology (GO) terms were then transferred to the sequences and eventually merged with existing GO terms resulting from mapping, InterProScan, EggNOG (evolutionary genealogy of genes: non-supervised orthologous groups) annotation and KEGG pathways [43], obtained with eggNOG-Mapper 1.0.3 with EggNOG 5.0.0 [42]. The remaining transcripts without annotation were further conducted to Rfam blast search against Xfam servers (rfam.xfam.org) [90]. Enrichment analyses to determine over/underexpressed pathways were performed to the categorical GO terms corresponding to the DEGs, excluding those transcripts corresponding to non-metazoans hits, and filtering the remaining GO terms of Bacteria, Virus, and other putative contaminants. The enriched genes/pathways were statistically determined with the hypergeometric distribution significance test (Fisher’s exact test, FDR < 0.05).

## Figures and Tables

**Table 1:** Statistics summary of transcriptomic analyses performed with wild cockles sampled in Ria de Aveiro, Portugal, exposed to Diarrhetic Shellfish Toxins (DSTs), and without DST. The table includes Raw Data Statistics from sequencing process, the de novo Assembly and Annotation metrics.

Summary of Analyses per Sample/Condition ^1^	Statistics	Ce_DGc	Ce_DGe	Ce_Gc	Ce_Ge
**Raw Data Statistics**	Total Bases ^2^	6,248,071,054	6,402,268,630	6,258,018,330	6,333,078,920
	Read Count ^3^	41,377,954	42,399,130	41,443,830	41,940,920
	GC (%) ^4^	42.33	41.63	41.09	41.05
	AT (%) ^5^	57.67	58.37	58.91	58.95
	Q20 (%) ^6^	97.95	97.89	98.02	97.9
	Q30 (%) ^7^	94.06	93.83	93.98	93.8
**de novo** **Transcriptome Assembly (Trinity v2.10.0)**	Assembled transcripts	684,723			
	Total genes	361,460.0			
	Minimum/Maximum Contig length (bp: base pair) ^8^	200/42,164 bp			
	Contig length (N50) ^8^	1094 bp			
	Contig length (Average) ^9^	736.09 bp			
	GC (%)	37.39			
	Total assembled bases (all transcripts)	504,019,879			
	Total assembled bases (longest isoform per gene)	199,701,724			
	Percentage of all read aligned/not aligned ^10^	92.1%/7.8%	92.4%/7.5%	93.1%/6.8%	93.3%/6.6%
**Assembly Completeness (BUSCO v5) ^11^**	number of BUSCOs/species	954/65			
	Complete Single-Copy	282/29.56%			
	Complete duplicated	658/69%			
	Fragmented	8/0.84%			
	Missing	6/0.63%			
**Assembly Clustering ^12^ (CD-HIT 4.8.1) and** **Completeness ^11^**	number of clusters at 90% identity	402,961			
	Complete Single-Copy	771/80.82%			
	Complete Duplicated	170/17.82%			
	Fragmented	8/0.84%			
	Missing	5/0.52%			
**ORFs prediction ^13^ (TransDecoder v5.5.0)**	Transcripts with ORF (best isoform per gene)	48,107			
**ORFs Annotation**	Blast Hits nr/NCBI (Metazoa/non-Metazoa) ^14^	37,184/362			
	Blast Hits SwissProt (Metazoa/non-Metazoa) ^15^	26,968/22			
	Averall ORFs with Blast hits/no hits ^16^	37,262 (77.5%)/10,845 (22.5%)			
	EggNOG ^17^	31,848 (66.2%)			
	KEGG Pathways/KO ^18^	12,760 (25.9%)/20,621 (42.8%)			
	IPS/GO annotation (%) ^19^	48,107 (100%)/19,398 (40.3%)			
	Gene Ontology (ORFs with GO annotation) ^20^	21,799 (45.3%)			

^1^ Sample ID/Condition: **Ce_DGc**: *Cerastodesma edule* digestive gland (DG) without dinophysistoxins (DSTs), **Ce_DGe**: *C. edule* DG exposed to DSTs, **Ce_Gc**: *C. edule* gills (G) without DSTs, **Ce_Ge**: *C. edule* gills exposed to DSTs. ^2^ Total read bases: Total number of bases sequenced. ^3^ Total reads: Total number of reads, corresponding to the sum of read 1 (Upstream Files Pattern: _1) and read 2 (Downstream Files Pattern: _2), obtained from Illumina paired-end sequencing. ^4^ GC(%): GC content. ^5^ AT(%): AT content. ^6^ Q20(%): Ratio of bases that have Phred quality score (expresses the accuracy of each nucleotide) of over 20 (99% base call accuracy). ^7^ Q30(%): Ratio of bases that have Phred quality score (expresses the accuracy of each nucleotide) of over 30 (99.9% Base call accuracy). ^8^ N50: is defined as the sequence length of the shortest contig at 50% of the total transcriptome/genome length. ^9^ Average contigs length. ^10^ RNA-Seq Read Representation corresponding to the percentage of the overall alignment (reads aligned/not aligned). ^11^ The de novo assembly quality and completeness assessment with the Benchmarking Universal Single-Copy Orthologs (BUSCOs), showing the number of BUSCOs/relative representation (percentage) of Complete Single-Copy, Complete Duplicated, Fragmented and Missing. BUSCO v5 run in mode: Transcriptome, lineage Metazoa, e-value 1.0E−3. ^12^ Sequence redundancy of the de novo assembled transcriptome was reduced using CD-HIT 4.8.1, setting 0.9 as the Sequence Identity Threshold. ^13^ Protein Coding Sequences (CDS) obtained by six-frame translation with TransDecoder v5.5.0., considering a minimum length of 100 amino acids for open reading frames (ORFs), homology to known proteins via Pfam searches, and the best/longest isoform per gene. ^14^ Blast hits of the 48,107 CDS (best isoforms) obtained from the transcriptome assembly searched against the non-redundant (nr) protein database from NCBI, using BlastX program, filtering by the taxonomic section Metazoa/non-Metazoans (accessed on 11 May 2021), setting a cut-off e-value 1.0E−3. ^15^ Blast hits of the 48,107 CDS (best isoforms) obtained from the transcriptome assembly searched against the protein database UniProtKB/Swiss-Prot, using BlastX program, filtering by the taxonomic section Metazoa/non-Metazoans (accessed on 12 May 2021), setting a cut-off e-value 1.0E−3. ^16^ Summary of Blast hits/no hits found against both databases searched, and their relative representation (percentage) within all (48,107) predicted ORFs. ^17^ Functionally annotated ORFs according to orthologous groups (OGs: i.e., COGs, arCOGs, ENOGs, KOGs), using the eggNOG Mapper 1.0.3 with EggNOG 5.0.0, and its relative representation (percentage) within all (48,107) predicted ORFs. ^18^ Functionally annotated ORFs according to Kyoto Encyclopedia of Genes and Genomes (KEGG) pathways and KEGG Orthology (KO), and its relative representation (percentage) within all (48,107) predicted ORFs. ^19^ Functionally annotated ORFs according to Gene Ontology (GO) and known proteins domains using InterProScan (IPS) v5.51–85.0, searched against InterPro member databases (i.e., Pfam, PROSITE, PRINTS, ProDom, SMART, TIGRFAMs, PIRSF, SUPERFAMILY, Gene3D, and PANTHER), as well as its relative representation (percentage) within all (48,107) pred. ^20^ Overall functionally annotated ORFs according to Gene Ontology (GO) and its relative representation (percentage) within all (48,107) predicted ORFs.icted ORFs.

**Table 2:** List of Differentially Expressed Genes (DEGs) related to known biomarkers involved in the shellfish molecular response after DSTs exposure.

Functional ^1^ Categories	Gene/Transcript ^2^	Gene/Transcript Description ^3^	Gene/Transcript ^4^ GO/IP Description	Organism ^5^ (Accession)	DEGs Subset ^6^ (UP-Regulated/Down-Regulated) LFC ^7^					
					Unique (DG) ^6.1^ 185 UR	Unique (DG) ^6.2^ 168 DR	Common 16 UR ^6.3^	Common 189 DR ^6.4^	Unique (G) ^6.5^ 80 UR	Unique (G) ^6.6^ 456 DR
Antioxidant system	TRINITY_DN158227_c0_g1	Extracellular superoxide dismutase [Cu-Zn]	Superoxide metabolic process; metal ion binding	*Fasciolopsis buski* **(KAA0197766.1)**		−8.15				
	TRINITY_DN250845_c0_g1	Extracellular superoxide dismutase [Cu-Zn]		*Fasciolopsis buski* **(KAA0197766.1)**		−8.15				
	TRINITY_DN57639_c0_g2	Superoxide dismutase [Cu-Zn]		*Elysia chlorotica* **(RUS83892.1)**						−9.14
	TRINITY_DN54684_c0_g1	Glutathione peroxidase	Response to oxidative stress; glutathione peroxidase activity	*Clonorchis sinensis* **(GAA50772.1)**						−7.91
	TRINITY_DN1482_c1_g1	Glutathione hydrolase 1 proenzyme-like	Glutathione catabolic process glutathione hydrolase activity	*Pecten maximus* **(XP_033752752.1)**	2.84					
	TRINITY_DN7416_c0_g1	Glutathione S-transferase Mu 3	Glutathione metabolic process; protein binding	*Alligator mississippiensis* **(XP_006260118.1)**						−8.29
	TRINITY_DN8990_c0_g1	Putative glutathione-specific γ-glutamyl cyclotransferase 2	Glutathione catabolic process; γ-glutamyl cyclotransferase activity	*Octopus vulgaris* **(XP_029637948.1)**	3.46					
Metabolic detoxification	TRINITY_DN9032_c0_g3	Cytochrome P450 2U1-like	Monooxygenase activity; iron ion binding; oxidoreductase activity Oxidoreductase activity, acting on paired donors, with incorporation or reduction of molecular oxygen; heme binding	*Mizuhopecten yessoensis* **(XP_021373153.1)**	3.66					
	TRINITY_DN2028_c0_g1	Steroid 17-α-hydroxylase /17,20 lyase-like		*Pecten* *maximus* **(XP_033763830.1)**			5.2			
							3.1			
	TRINITY_DN12127_c0_g1	ABC transporter G family member 21-like isoform X1	ATPase-coupled transmembrane transporter activity	*Pomacea canaliculate* **(XP_025089510.1)**	6.64					
	TRINITY_DN86_c1_g1	Phosphatidylcholine translocator ABCB4-like	ATP binding; xenobiotic transmembrane transporter activity	*Mizuhopecten yessoensis* **(XP_021367043.1)**	3.18					
Transcription	TRINITY_DN70033_c0_g1	KH domain protein	RNA processing; DNA binding; RNA binding	*Opisthorchis felineus* **(TGZ67600.1)**				−8.01		
								−9.24		
	TRINITY_DN22122_c0_g1	Cell division cycle and apoptosis regulator protein 1	regulation of transcription, DNA-templated	*Paragonimus westermani* **(KAA3674315.1)**				−9.21		
								−10.38		
	TRINITY_DN54844_c0_g2	CREB-binding protein	regulation of transcription, DNA-templated; protein binding	*Schistosoma japonicum* **(TNN09875.1)**						−7.39
	TRINITY_DN116751_c0_g1	Transcription elongationregulator 1	FF domain; protein-protein interaction	*Paragonimus westermani* **(KAA3677206.1)**				−8.26		
								−8.56		
cytoskeleton, actin related proteins	TRINITY_DN9687_c0_g1	Tubulin α chain testis- specific	structural constituent of cytoskeleton; GTP binding; microtubule	*Fasciola hepatica* **(KAA3677206.1)**				−8.65		
								−8.66		
	TRINITY_DN9687_c1_g2	Tubulin α-chain		*Fasciola hepatica* **(THD25403.1)**						−8.48
	TRINITY_DN181044_c0_g1	Actin depolymerizing factor	actin filament depolymerization; actin binding	*Opisthorchis viverrine* **(XP_009175913.1)**						−7.5
	TRINITY_DN34498_c0_g1	Actin-1 isoform X2	actin family	*Rattus norvegicus* **(XP_002726247.1)**						−7.08
	TRINITY_DN613_c1_g1	VChain V, beta-actin	ATP binding; cytoplasm; cytoskeleton	*Sus scrofa* **(pdb|5NW4|)**				−11.08		
								−11.81		
	TRINITY_DN6932_c0_g1	Actin β/γ 1	Actin family	*Clonorchis sinensis* **(GAA57823.1)**				−8.61		

	TRINITY_DN7730_c0_g1	Actin β/γ 1		*Opisthorchis felineus* **(TGZ74009.1)**				−5.42		
								−7.54		
Inmune response	TRINITY_DN10313_c0_g2	Complement C1Q-like protein	Protein binding	*Sinonovacula constricta* **(QCO31675.1)**		−3.83				
	TRINITY_DN1050_c0_g1	Complement C1q tumor necrosis factor-related protein 4-like		*Octopus vulgaris* **(XP_029647271.1)**	8.06					
	TRINITY_DN14010_c0_g1	Putative C1q domain containing protein MgC1q83		*Mytilus galloprovincialis* **(CBX41732.1)**						−8.38
	TRINITY_DN1909_c0_g2	Complement C1q-like protein 2		*Lates calcarifer* **(XP_018543622.1)**	3.91					
	TRINITY_DN45102_c0_g1	Complement C1Q-like protein		*Pecten maximus* **(XP_033749093.1)**		−5.41				
Heat shock protein HSP 90 (HSP90)	TRINITY_DN16801_c0_g1	Hsp90 protein	Protein folding; ATP binding; ATP hydrolysis activity; unfolded protein binding	*Opisthorchis viverrine* **(XP_009172981.1)**				−8.31		
								−10.24		
	TRINITY_DN260239_c0_g1	Hsp90 co-chaperone Cdc37 isoform 1	Protein kinase binding	*Schistosoma japonicum* **(AAP06244.1)**						−7.99

^1^ Major Functional Categories related to known proteins/biomarkers involved in dinophysistoxins (DSTs) metabolism and detoxification. ^2^ Sequence name of the DEGs identified within the corresponding Major Functional Category. ^3^ Blast top-hit description of the corresponding DEGs. ^4^ Gene Ontology (GO)/InterProScan (IPS) description of the corresponding DEG (Gene/transcript). ^5^ Organism and accession number of the corresponding Blast top-hit obtained for each DEGs. ^6^ Subsets of unique and overlapped DEGs (used as test set in the enrichment analyses), identified in the digestive gland and gills of the cockle *C. edule*, exposed to DSTs, where contrast condition and reference/control sample were Ce_DGe/Ce_Ge and Ce_DGc/Ce_Gc, respectively. Subsets: ^6.1^ 185 unique up-regulated DEGs identified in the digestive gland (DG) (unique DG 185 UR, Ce_DGe_vs_Ce_DGc); ^6.2^ 168 unique down-regulated DEGs identified in the digestive gland (unique DG 168 DR, Ce_DGe_vs_Ce_DGc); ^6.3^ 16 common DEGs with a similar up-regulated pattern identified in both conditions exposed to DSTs (common 16 UR); ^6.4^ 189 common DEGs with a similar down-regulated pattern identified in both conditions exposed to DSTs (common 189 DR); ^6.5^ 80 unique up-regulated DEGs identified in the gills (Ce_Ge_vs_Ce_Gc, unique (G) 80 up-regulated); ^6.6^ 456 unique down-regulated DEGs as test set (file: test_set_456_unique_DEGs_down_Ce_Ge_vs_Ce_Gc). ^7^ The log-fold change (M) and the absolute value of the difference in expression obtained with NOISeq R/Bioc package between conditions: Ce_DGe_vs_Ce_DGc, in the Digestive Gland (DG), and Ce_Ge_vs_Ce_Gc in the Gills (G).

## Data Availability

The transcriptomic project has been registered with the BioProject database under BioProject ID: PRJNA739261 (http://www.ncbi.nlm.nih.gov/bioproject/739261). The pre-processed raw data have been deposited at the NCBI Sequence Read Archive (SRA, http://www.ncbi.nlm.nih.gov/Traces/sra), under accessions SAMN19778280 (Ce_DGc, https://www.ncbi.nlm.nih.gov/biosample/19778280), SAMN19778281 (Ce_DGe, https://www.ncbi.nlm.nih.gov/biosample/19778281), SAMN19778282 (Ce_Gc, https://www.ncbi.nlm.nih.gov/biosample/19778282), SAMN19778283 (Ce_Ge, https://www.ncbi.nlm.nih.gov/biosample/19778283). The Transcriptome Shotgun Assembly project has been deposited at DDBJ/EMBL/GenBank under the accession GJGL00000000. DATASET S1: The resulting original files obtained from de novo transcriptome assembly and clustering analyses (e.g., .fasta and .gff files, transcript_to_gene_map.txt, summary files), have been deposited at the Mendeley Data repository as DATASET S1 (http://dx.doi.org/10.17632/vkw2jcmmzt.1). DATASET S2: The resulting .fasta and .gff files corresponding to the open reading frames (ORFs) prediction obtained, as well as summary files have been deposited at the Mendeley Data repository as DATASET S2 (http://dx.doi.org/10.17632/bgps766mxp.1). DATASET S3: All data used for the gene expression calculation comprising the count tables, as well as the resulting files obtained from the differential expression analyses, have been deposited at the Mendeley Data repository as DATASET S3 (http://dx.doi.org/10.17632/6z6rpf9w8z.1). DATASET S4: The additional files used for enrichment analyses have been deposited at the Mendeley Data repository as DATASET S4 (http://dx.doi.org/10.17632/5yp5ncm2vp.1).

## Supplementary Materials

The following are available online at https://www.mdpi.com/article/10.3390/toxins13110784/s1, Figure S1: The de novo assembly quality and completeness assessment with the Benchmarking Universal Single-Copy Orthologs (BUSCOs), Figure S2: The Protein Coding Sequences (CDS) obtained by six-frame translations with TransDecoder v5.5.0., considering a minimum length of 100 amino acids for open reading frames (ORFs) with homology to known proteins via Pfam searches, and the best/longest isoform per gene, Figure S3: Species distribution according to Orthologous Groups (OGs) categories obtained from the eggnog database using the eggNOG-Mapper 1.0.3 with Egg-NOG 5.0.0, Figure S4: Distribution of counts per gene across libraries (based on a count-per-million basis (CPM), Figure S5: Multi-dimensional scaling (MDS) plot of the Differential Expressed Genes (DEGs), Table S1: Transcripts used for pairwise differential expression analyses, Table S2: Putative contaminants filtered before pairwise differential expression analyses, Table S3: Differential Expressed Genes (DEGs) obtained from the pairwise comparison between the condi-tions studied in the transcriptomic profile of the cocke Cerastoderma edule exposed to Diarrhetic Shellfish Toxins (DSTs), using NOISeq v2.30.0, with 5 technical replicates simulated per sample and *p*-value 0.01, Table S4: Venn Diagram results between the Differentially Expressed Genes (DEGs) from the pairwise comparison between the tested condition Ce_DGe_vs_Ce_DGc and Ce_Ge_vs_Ce_Gc, obtained in the transcriptomic analyse of the cockles Cerastoderma edule, Table S5: Enrichment analyses performed to the Differentially Expressed Genes (DEGs) using the hypergeometric distribution significance test (Fisher’s exact test, FDR < 0.05), Table S6: results of the Venn Diagram performed to 23 common enriched GO terms in the 189 shared downregulated DEGs from the digestive gland, and the subset of 456 unique DEGs downregulated in the gills.
