# Supplementary material for: Transcriptomic Profile of the Cockle Cerastoderma edule Exposed to Seasonal Diarrhetic Shellfish Toxin Contamination
Source: Toxins (Basel). 2021 Nov 5;13(11):784. doi: 10.3390/toxins13110784 (PMC8625317; doi:10.3390/toxins13110784)
Supplement: Supplementary file 1 [file toxins-13-00784-s001.zip › Supplementary material.pdf]

Supplementary Materials: Transcriptomic profile of the cockle *Cerastoderma edule* exposed to seasonal Diarrhetic Shellfish Toxins outbreaks

Dany Domínguez-Pérez, José Carlos Martins, Daniela Almeida, Pedro Reis Costa, Vitor Vasconcelos, Alexandre Campos

A)

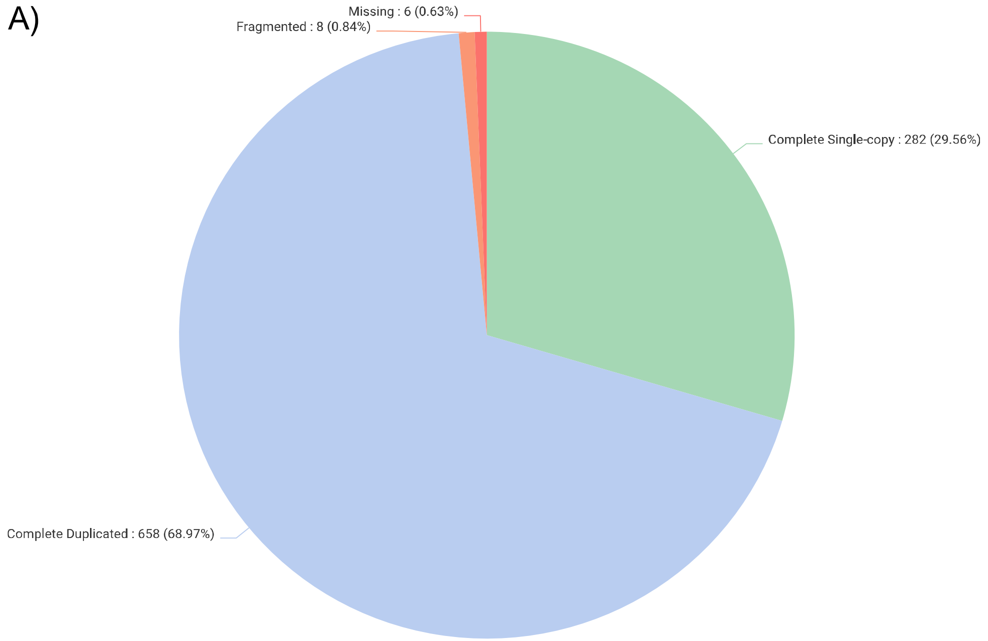

B)

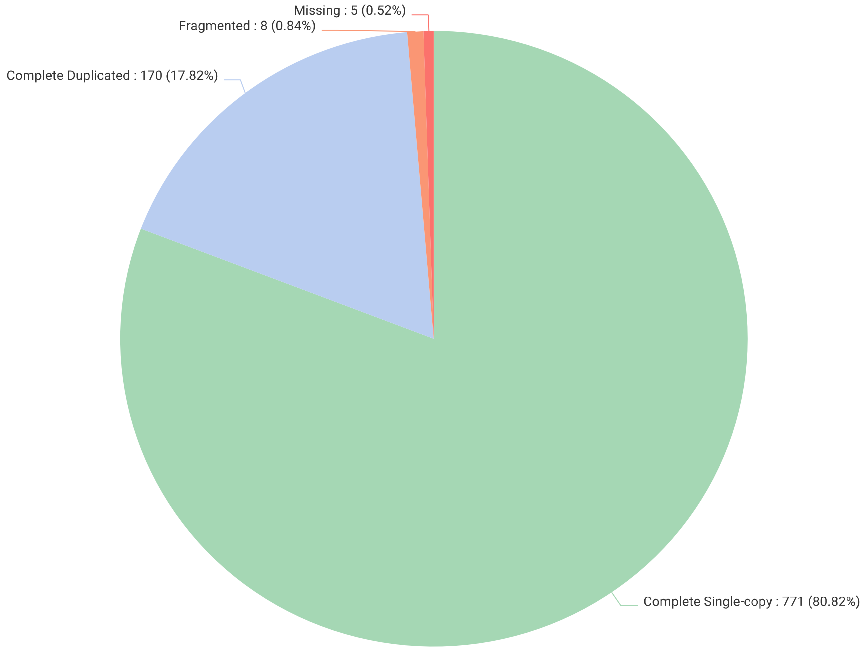

Figure S1: The de novo assembly quality and completeness assessment with the Benchmarking Universal Single-Copy Orthologs (BUSCOs). The figure shows the number of BUSCOs/relative representation (percentage) of Complete Single-Copy, Complete Duplicated, Fragmented and

Missing transcripts/genes A) BUSCOs of the de novo transcriptome assembly of the wild cockle *C. edule*; B) BUSCOs of the transcriptome clusters obtained at 0.9 of sequence identity, both run with BUSCO v5 run in mode: Transcriptome, lineage Metazoa, e-value 1.0E-3.

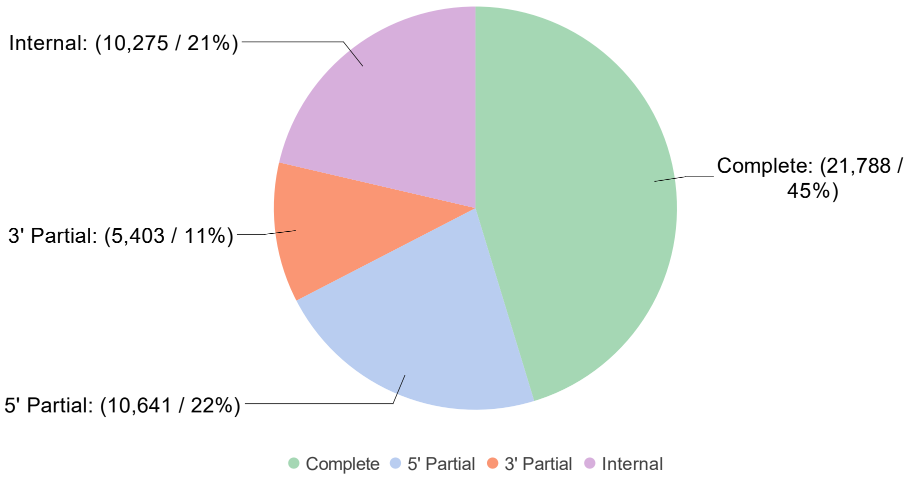

Figure S2: The Protein Coding Sequences (CDS) obtained by six-frame translations with TransDecoder v5.5.0., considering a minimum length of 100 amino acids for open reading frames (ORFs) with homology to known proteins via Pfam searches, and the best/longest isoform per gene.

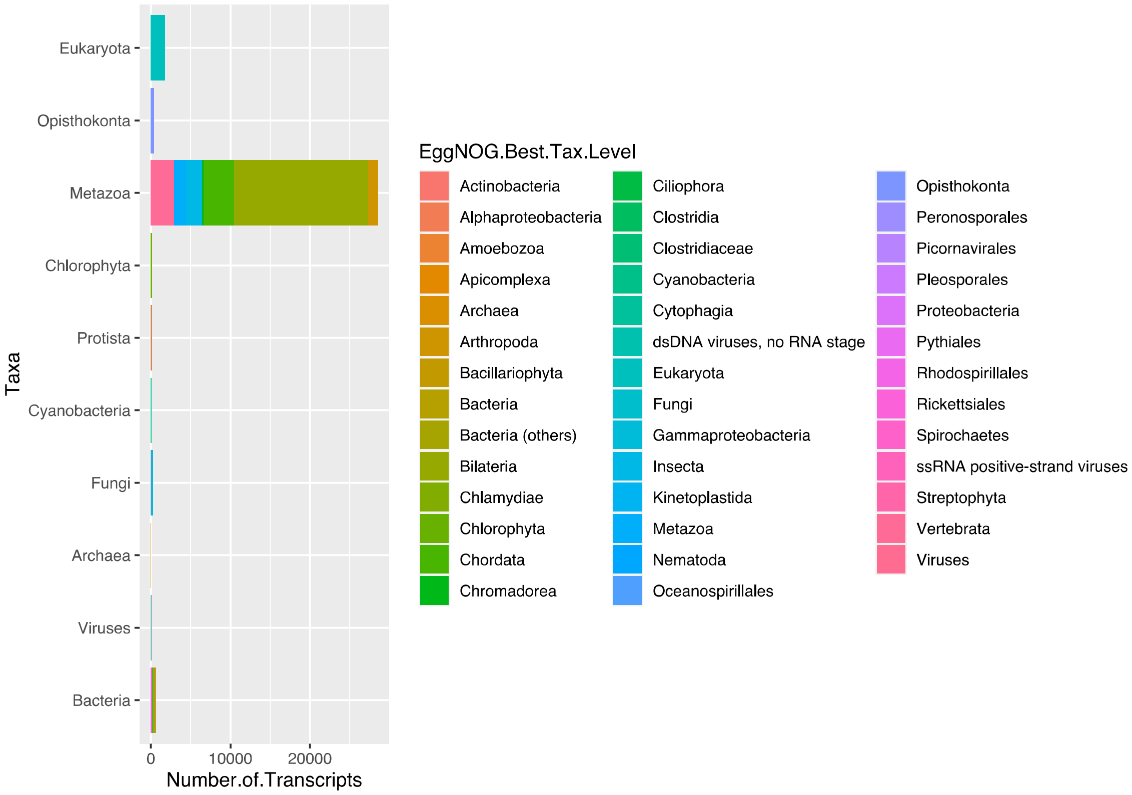

Figure S3: Species distribution according to Orthologous Groups (OGs) categories obtained from the eggnog database using the eggNOG-Mapper 1.0.3 with EggNOG 5.0.0. The figure shows the number of transcripts (x-axis) found per major Taxa (y-axis), identified within the 48,107 open reading frames (ORFs) as best/longest isoform per gene predicted from the de novo transcriptome assembly of *C.*

edule. It should be noted that some transcripts/genes corresponding to different taxonomic level (EggNOG.Best.Tax.Level), were grouped in the same bar as the hits obtained for metazoa lineage, rather than other non-metazoans organisms.

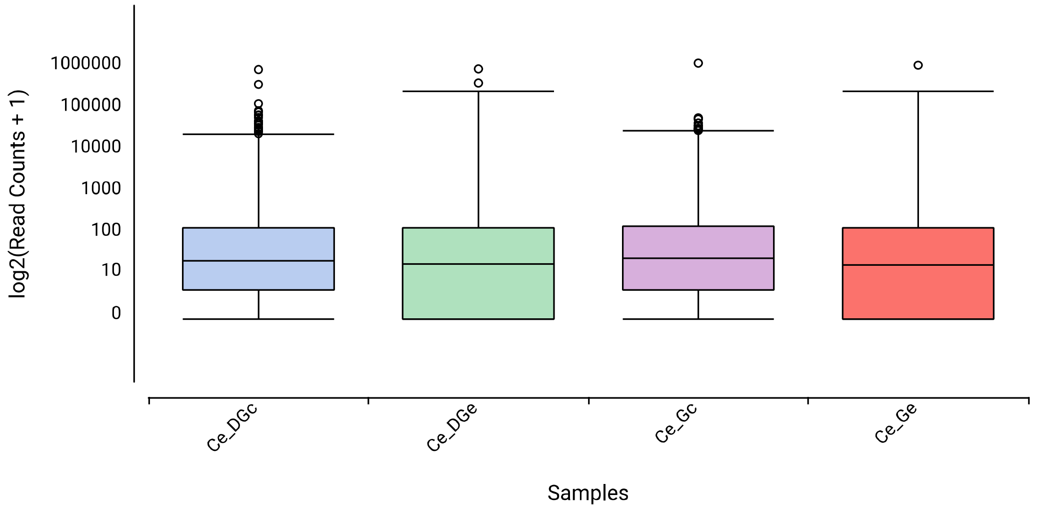

Figure S4: Distribution of counts per gene across libraries (based on a count-per-million basis (CPM). The figure shows the normalized counts per sample (x-axis), as the log2 number of counts per coding gene (y-axis). The corresponding samples exposed to Diarrhetic Shellfish Toxins (DSTs) are designated as Ce_DGe and Ce_Ge, in the digestive gland and gills of the cockles *C. edule*, respectively, whereas Ce_DGc and Ce_Gc depict the samples without DSTs in both organs, in the same order.

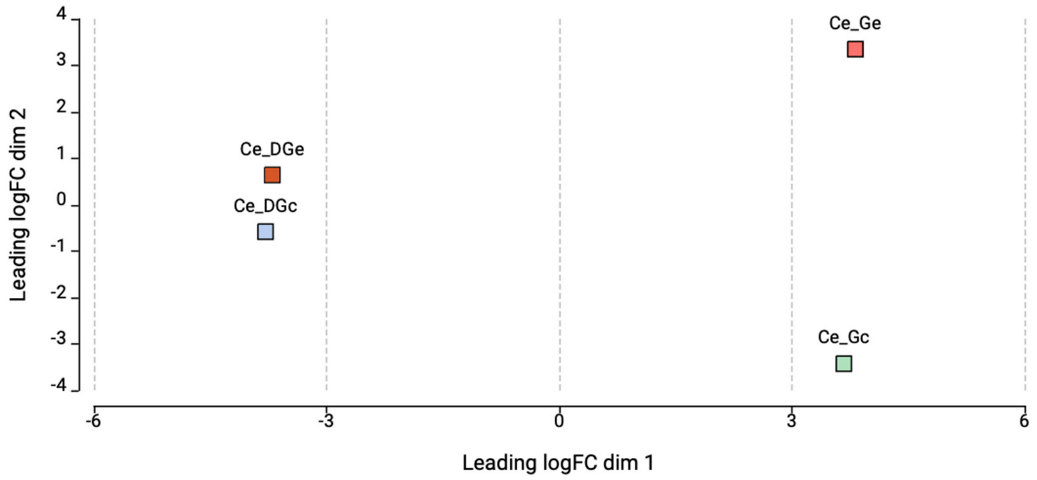

Figure S5: Multi-dimensional scaling (MDS) plot of the Differential Expressed Genes (DEGs). The figure displays the similarity between the samples of both tissue and conditions studied, in which distances correspond to leading log-fold-changes between the DEGs of each pairwise comparison (digestive glands: Ce_DGe_versus_Ce_DGc and gills: Ce_Ge_versus_Ce_Gc). The corresponding samples exposed to Diarrhetic Shellfish Toxins (DSTs) are designated as Ce_DGe and Ce_Ge for the digestive gland and gills of the cockles *C. edule*, in the same order, whereas Ce_DGc and Ce_Gc depict the samples without DSTs, respectively.
